# “Therapy Beyond the Screen”: A Qualitative Exploration of Tele-Therapy Experiences Among Clinical Psychologists and Speech and Language Therapists

**DOI:** 10.3390/healthcare14060716

**Published:** 2026-03-11

**Authors:** İbrahim Can Yaşa, Pınar Akgün, Sakine Deniz Yılmaz, Muhsin Dölek, Seda Eyilikeder Tekin, Ayşe Serra Kaya

**Affiliations:** Speech and Language Therapy Department, Faculty of Health Sciences, Bahcesehir University, Istanbul 34353, Turkey

**Keywords:** teletherapy, clinical psychology, speech and language therapy, qualitative research, Türkiye, therapeutic alliance, digital health, healthcare practitioners, cross-professional comparison

## Abstract

**Highlights:**

**What are the main findings?**

- This qualitative study examines the experiences of clinical psychologists and speech and language therapists in Türkiye who deliver online therapy, with a focus on teletherapy.
- Findings indicate that both professional groups regard online therapy as an effective option because it increases accessibility, provides time savings, and enables therapeutic services to be delivered independent of location.

**What are the implications of the main findings?**

- While clinical psychologists and speech and language therapists reported similar experiences in relation to the therapeutic relationship, the techniques and materials used, ethical considerations, and perceived advantages and limitations, they also demonstrated approaches that diverged in profession specific practices.
- Participants emphasized that challenges encountered during online therapy point to areas requiring improvement rather than diminishing the method’s effectiveness, and overall, the findings suggest that teletherapy may become a more widespread and effective intervention modality as technological infrastructure strengthens and therapists gain greater experience with online delivery.

**Abstract:**

**Background/Objectives:** With the diversification of technological tools, the prevalence of online therapy has increased among both clients and practitioners. Clinical psychologists and specialist speech and language therapists are two professional groups that frequently use online therapy. Understanding their experiences is important for evaluating the effectiveness of this service model and informing its further development. The aim of this study was to examine and compare the online therapy experiences of these two professional groups. **Methods:** This qualitative study included 10 practitioners (five clinical psychologists and five specialist speech and language therapists) who were actively delivering online sessions in Türkiye. Participants were recruited using convenience sampling. Data were collected through semi structured, open-ended interviews conducted online and were analyzed using thematic analysis. **Results:** A total of 8 themes emerged regarding practitioners’ experiences with online therapy, including the therapeutic relationship, techniques and materials used, advantages and disadvantages, coping strategies, professional reflections, required improvements, ethical issues, and recommendations. Similarities and differences were identified between the two professional groups across these themes. **Conclusions:** Participants considered online therapy to be an effective and feasible option, particularly due to increased accessibility, time savings, and the ability to deliver services independent of location. Challenges encountered during online therapy were described as areas requiring improvement rather than factors undermining effectiveness. Overall, the findings suggest that online therapy may become a more widespread intervention modality as technological infrastructure improves and practitioners gain greater experience with online delivery.

## 1. Introduction

### 1.1. Evolution and Technical Framework of Teletherapy

Teletherapy is an alternative and complementary service to face-to-face interventions that enables the delivery of services such as counseling, assessment, and intervention through information and communication technologies [1]. With the acceleration of digitalization, teletherapy practices have rapidly expanded due to restrictions on face-to-face services and have become an important alternative intervention modality in fields such as health care and psychotherapy [2,3,4,5]. This service model encompasses technologies such as computers, mobile devices, webcams, headset microphones, internet connectivity, videoconferencing software, and email or text messaging [6,7]. Among these, internet-based videoconferencing systems that allow bidirectional and synchronous audio visual communication are used most frequently [8].

### 1.2. Professional Standards and General Utility

Clinical psychologists (CPs) and speech and language therapists (SLTs) are two professional groups that have been highlighted as implementing this service model effectively [4,9]. In 2013, the American Psychological Association (APA) published the Guidelines for the Practice of Telepsychology, establishing standards for the remote delivery of psychotherapy services. Similarly, the American Speech Language Hearing Association [10] defines teletherapy as a model in which therapy services are delivered remotely via the internet and telecommunication technologies.

Teletherapy has been shown to be used effectively in both speech and language therapy practice and psychotherapy services [2,10,11]. Teletherapy has been implemented effectively in areas such as swallowing, fluency and speech sound disorders, depression, anxiety, and eating disorders [12,13,14,15]. While the literature highlights advantages of teletherapy such as reducing transportation barriers, increasing access to services, enabling long term follow up in the home environment, providing temporal flexibility, and offering a sustainable model [9,16,17,18], it also notes disadvantages, including connection problems, inadequate video or audio quality, and the potential for environmental distractors to negatively affect the therapeutic process [19,20]. In addition, clinical challenges have been reported, such as difficulties in establishing a trusting relationship, reduced naturalness of interaction, and impacts on the quality of the therapeutic process [21]. From an ethical perspective, confidentiality, data security, and the risk of unauthorized recording have emerged as key areas of concern.

### 1.3. Teletherapy in Clinical Psychology: Effectiveness and Ethical Dimensions

The effectiveness of online psychotherapy is multidimensional, involving process, outcome, and subjective experience. While structured models such as Cognitive Behavioral Therapy (CBT) and Acceptance and Commitment Therapy (ACT) [22,23] and therapist-guided interventions [24] yield outcomes comparable to face-to-face therapy, practitioners often report reservations about maintaining the therapeutic frame and emotional synchrony [3]. Challenges in rapport-building and the absence of eye contact can complicate self-expression, particularly in cases of depression [25]. Consequently, teletherapy may have limited efficacy for individuals with severe psychopathology, developmental disorders, or those under 18 [11].

From an ethical and practice-related perspective, risks concerning confidentiality, technical disruptions, and environmental control are more prominent in digital settings than in traditional ones [26,27]. Therefore, professional guidelines emphasize technical competence, secure infrastructure, and the rigorous assessment of client suitability to address these clinical, technical, and ethical dimensions in an integrated manner [28].

### 1.4. Teletherapy in Speech and Language Therapy: Empirical Evidence

Research confirms teletherapy as a robust alternative to face-to-face SLT, demonstrating comparable outcomes across stuttering, neurogenic, and swallowing disorders [29,30,31]. Clinicians report high competence in delivering online services [5], with specific suitability highlighted for voice and fluency disorders in Türkiye [18].

While teletherapy fosters family involvement, reduces costs, and shortens waiting lists, it presents logistical hurdles such as technology access and the absence of physical contact during assessment [20,21,32]. Despite these barriers, clinical attitudes have shifted positively, with parental satisfaction and motivation frequently exceeding therapist levels [5,33]. The rapid global expansion of teletherapy across both SLT and psychotherapy underscores its status as a primary intervention modality, making the exploration of practitioner experiences essential for refining service delivery [12,13,14,34,35,36].

### 1.5. Rationale and Aims of the Present Study

Nevertheless, some studies emphasize that face-to-face therapy may be more effective, particularly in cases involving severe psychopathology, complex presentations, or situations in which establishing a therapeutic alliance is challenging, and that the limitations of teletherapy should be taken into account [37,38]. Accordingly, examining the experiences of CPs and SLTs who actively use teletherapy is critical for enhancing the acceptability of the practice, therapeutic effectiveness, and professional satisfaction [39,40]. Studies that directly compare the teletherapy experiences of these two professional groups are also limited. For these reasons, the first aim of the present study was to examine in depth the teletherapy experiences of CPs and SLTs who actively use teletherapy, and the second aim was to compare the experiences of the two professional groups to identify similarities and differences.

### 1.6. The Present Study and Research Questions

The first aim of the present study was to examine in depth the teletherapy experiences of CPs and SLTs who actively use teletherapy, and the second aim was to compare the experiences of the two professional groups to identify similarities and differences. Based on this, the study seeks to answer the following research questions:

1. How do CPs and SLTs describe their subjective experiences and therapeutic relationship in teletherapy?
2. What are the specific technical and ethical challenges encountered by each profession?
3. How do practitioners from both fields perceive the future role of teletherapy within the Turkish healthcare context?

## 2. Methods

### 2.1. Ethical Approval

This study was approved by the Ethics Committee of Bahçeşehir University (Ethics Approval No: E-85646034-604.01-119878, Date: 3 December 2025).

### 2.2. Design

This study utilized a descriptive phenomenological design, a qualitative research approach rooted in the philosophical tradition of Husserl [41]. Descriptive phenomenology was specifically chosen as it allows for a rigorous exploration of the ‘essence’ of practitioners’ lived experiences without the imposition of pre-existing theoretical frameworks [42]. By employing this approach, the study aimed to capture the subjective meanings that Clinical Psychologists and SLTs assign to their teletherapy practices, thereby providing an in-depth understanding of the phenomenon from the participants’ own perspectives.

### 2.3. Participants

The study participants comprised 10 professionals (five specialist SLTs and five CPs), predominantly female (n = 9) and primarily based in Istanbul, Türkiye. Participants were recruited between February 2024 and May 2024 using a combination of convenience and purposive sampling. Recruitment invitations were disseminated through professional social media networks (e.g., LinkedIn, specialized WhatsApp groups for therapists) and national professional associations in Türkiye.

All participants held postgraduate degrees (Master’s or PhD). To provide a deeper professional profile, CPs reported specialized training in established therapeutic modalities such as Cognitive Behavioral Therapy (CBT), EMDR, and Schema Therapy, while SLTs held clinical certifications in specific intervention protocols for pediatric speech sounds, swallowing disorders, and early childhood communication. Their professional experience in teletherapy ranged from 1 to 4 years, providing a rich spectrum of perspectives from both early adopters with extensive case histories (e.g., over 2000 cases) and more recent practitioners.

Data saturation was operationalized as the point where the gathering of new data no longer sparked new theoretical insights or revealed new codes within the emergent themes. Saturation was monitored concurrently and independently for each professional group to ensure cross-professional rigor. Conceptual saturation was reached at the 4th interview for the SLT group and the 5th interview for the CP group. Although the total sample size (N = 10) is relatively small, it is consistent with the standards of descriptive phenomenology, which prioritizes the ‘depth’ and ‘essence’ of lived experience over statistical generalization (Creswell & Poth, 2018) [41]. Participant characteristics are presented in Table 1.

As presented in Table 1, participants ranged in age from 25 to 42 years, and all held a graduate degree. Although most participants were based in Istanbul, there were also participants from Eskişehir and Mersin. Years of professional experience ranged from 2.5 to 5 years in the SLT group and from 3 to 20 years in the CP group. The SLTs reported working in areas such as stuttering, language and speech disorders, articulation disorders, voice disorders, and developmental language disorder. The CPs reported providing services in adult, child, family, and couples therapy. Regarding the number of online therapy cases, SLTs reported between 100 and 300 cases, whereas CPs reported between 100 and 2000 cases. Notably, CP5 had the highest level of online experience, with 2000 online therapy cases. Overall, these data indicate diversity across participants in terms of professional experience, areas of practice, and the extent and frequency of online therapy delivery.

### 2.4. Data Collection and Analysis

The research team comprised an associate professor with teletherapy experience in speech and language therapy, two assistant professors, two lecturers, and a psychologist with teletherapy experience in psychology. Data were collected between 20 December 2025 and 5 January 2026 using a semi-structured interview guide by the first and second authors. To accommodate the geographic distribution of participants and ensure a private, comfortable environment, all interviews were conducted online via the Zoom platform.

The first author (an SLT) conducted the interviews with SLTs, whereas the second author (a psychologist) conducted the interviews with CPs to mitigate power dynamics and enhance rapport through professional shared context. The interview guide was developed following a review of the relevant literature [20,21] and focused on four primary areas: (1) the transition to online practice, (2) perceived therapeutic relationship and clinical effectiveness, (3) technical and ethical challenges, and (4) professional outcomes. These categories were specifically designed to map onto the study’s research questions and the subsequent thematic analysis. The full interview questions list is provided as Supplementary Material S1.

Before the interviews, participants were informed about the study aim and procedures, and they were notified that interviews would be audio-recorded. Interviews were scheduled at mutually convenient times and lasted between 20 and 40 min.

All interviews (N = 10) were transcribed verbatim, yielding 2070 lines of text (63 pages). MAXQDA was used to support data management and analysis. Data were analyzed using a structured, iterative process: familiarization with the data, initial coding, theme development, reviewing and refining themes, defining and naming themes, and reporting the findings [43]. Coding was conducted using a hybrid approach that combined deductive and inductive strategies: an initial deductive coding framework was informed by the interview guide and the relevant literature, while additional codes were generated inductively from the data during iterative transcript review. All authors read the transcripts in detail. Codes developed through this process were compared and discussed until consensus was reached. Themes were developed by clustering conceptually related codes, and the coding and theme development procedures were reviewed by the research team to enhance analytic rigor [41,44]. Throughout the process, regular team discussions informed ongoing refinements to improve both the analytic clarity and the presentation of the results.

Several strategies were used to enhance the trustworthiness of the findings. Direct quotations were included to support interpretations. In addition, an expert with qualitative research experience, independent of the research team, reviewed the study procedures and analytic outputs. As a result of the analysis, 8 themes were identified and elaborated to enable an in-depth examination of the findings. Two case-based comparison models were also created in MAXMaps to examine how codes within the emergent themes were represented across the SLTs and CPs groups. The findings are presented in the relevant section and supported.

### 2.5. Reflexivity and Trustworthiness

The researchers acknowledged their roles as practitioners and academics within the fields of SLT and psychology. To mitigate potential power dynamics and social desirability bias, ‘profession-matched’ interviewing was employed: SLT participants were interviewed by an SLT researcher, and CP participants by a psychologist. This approach facilitated a shared professional language and rapport. During the coding process, any disagreements between the primary coders were resolved through ‘peer debriefing’ sessions with the senior authors until 100% consensus was reached. While inter-coder reliability (ICR) was not statistically calculated, consistency was ensured through a continuous iterative review of the codebook against the entire dataset.

## 3. Results

The frequency counts presented in the tables indicate the prevalence of specific codes among participants. However, these frequencies were utilized as a navigational tool rather than a measure of thematic importance; the analytical weight was primarily determined by the depth, nuance, and contextual richness of the participants’ narratives.

The findings of the study are presented across 8 themes, comparing the SLT and CP groups and supported by direct quotations.

### 3.1. Therapeutic Relationship in Online Therapy

**Figure 1:**
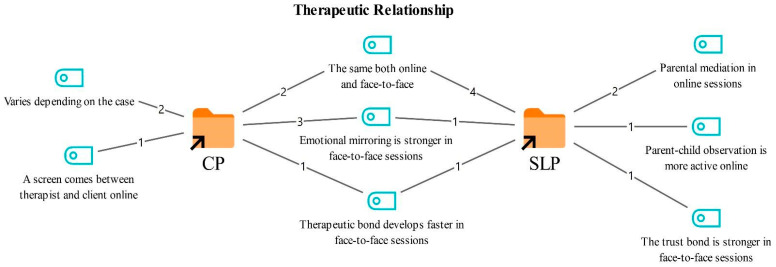
Codes related to the therapeutic relationship theme.

Participants were asked to compare their therapeutic relationship with clients in online therapy with that in face-to-face therapy. The resulting codes and their frequencies are presented in Figure 1.

According to Figure 1, the views of the SLT and CP groups converged on some shared codes while diverging on others. The shared codes highlighted by both professional groups included the similarity between online and face-to-face therapeutic relationships (6), stronger affective expression in face-to-face therapy (4), and faster establishment of the therapeutic bond in face-to-face settings (2). These codes were articulated by both groups and are positioned centrally in the figure.

*“The therapeutic relationship is essentially between the therapist and the client, so whether it is online or face to face does not create a very, very significant effect. In other words, we can maintain a therapeutic relationship with the client quite well in an online format.”*

*(SLT2)*

*“In face to face sessions, there is contact and emotional transfer. We also grasp things in more detail, for example leg movements or foot tapping, but online we can miss these.”*

*(CP4)*

*“I think face to face leads to a therapeutic relationship that progresses faster. Online feels a bit slower to me because there is a screen in between. I think there is a problem related to emotional transmission in online therapy.”*

*(CP1)*

In addition, the CPs noted that the quality of the therapeutic relationship may vary depending on the case (2) and that the screen can function as a barrier in the online format (1). In contrast, the SLTs emphasized that the online process often needs to be carried out through a parent (2), and that parent child observation becomes more active in this context (1). They also highlighted that the sense of trust may be stronger in face-to-face settings (1).

*“In some cases, face to face sessions can be more effective or more important in terms of establishing the relationship. So, while each case is unique, depending on the client’s needs, face to face sessions can sometimes allow the therapeutic relationship to be established more closely.”*

*(CP2)*

*“Because in face to face therapy, the family can directly observe that the therapist is approaching the child in a way that is specific to the child’s individual differences, how the child is greeted at the door, or even if the client is an adult or an older person. At least, I observed that face to face communication is stronger and that the bond of trust is more firmly established.”*

*(SLT1)*

### 3.2. Techniques/Approaches and Materials in Online Therapy

**Figure 2:**
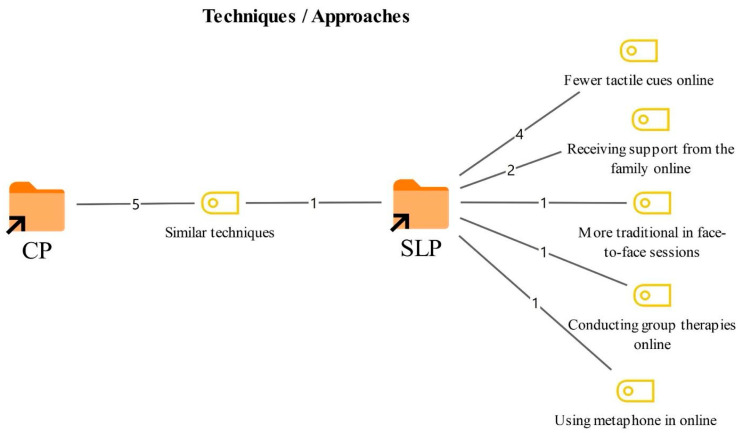
Codes related to the techniques/approaches theme. Note: this figure illustrates the hierarchical structure of the coding process and the conceptual map of the identified themes. The central nodes represent the primary themes, while the peripheral nodes indicate the sub-themes. The connections between nodes demonstrate the thematic flow and the conceptual overlap identified during the iterative analysis.

**Figure 3:**
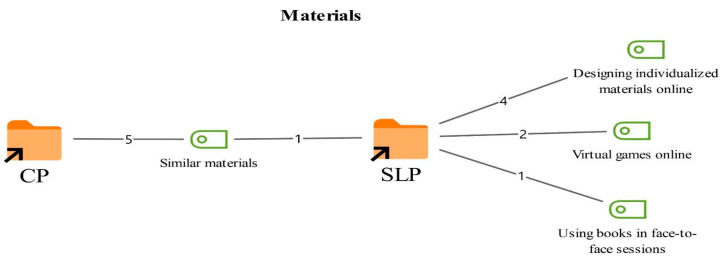
Codes related to the materials theme. Note: this figure highlights the distribution of themes across Clinical Psychologists (CP) and Speech and Language Therapists (SLT). While frequency counts (n) indicate the prevalence of specific codes within the data, the thematic weighting and prioritization were determined based on the qualitative richness and interpretive depth of the participants’ narratives, rather than statistical frequency alone.

Participants were asked whether the techniques/approaches and materials they used during online therapy differed from those used in face-to-face therapy, and to explain the nature and direction of any differences. The resulting codes and their frequencies are presented in Figure 2 and Figure 3.

As shown in Figure 2, the most prominent shared view was that similar techniques are used in both online and face-to-face therapy (6). Participants noted that core techniques remain applicable across both formats, although some differences may emerge during implementation.

*“I provide EMDR therapy, I use it. We can use EMDR online. In terms of techniques, we can do the same things, like tapping on the shoulder or tapping on the legs. If someone is not receiving EMDR, then they are not. If the person does not want to receive the intervention in some way, they will not. It is the same online and face to face. Perhaps we can demonstrate bodily and visual forms of intervention.”*

*(CP3)*

In contrast, the SLTs reported more pronounced changes in the use of certain techniques during online therapy. They indicated that tactile cues are used less frequently online (4), the need to obtain support from families increases in online therapy (2), and traditional approaches are more often preferred in face-to-face therapy (1). In addition, participants noted that the online format can facilitate group therapy (1) and that using metaphon in online therapy is possible (1).

*“If there is a situation where we need to directly touch the person, such as oral motor exercises, that is where it differs the most, I think, because we cannot touch them in online therapy. In those moments, as I mentioned earlier, I get support from families.”*

*(SLT2)*

*“When it is online, it becomes more difficult to provide tactile cues. Because of that, I use the therapy method I frequently use in a different way online. For example, if a traditional approach is used for speech sound disorders face to face, I use metaphon online… In online sessions, for instance, I can bring different people together using the Zoom application, and for desensitization in the same way. In that respect, I sometimes think it can be more beneficial.”*

*(SLT1)*

As shown in Figure 3, the shared view was that the same materials are used in both online and face-to-face therapy, although they are sometimes implemented through different adaptations (6). Participants emphasized that core materials do not fundamentally change, but that the online format may require certain adjustments.

*“I don’t think there is a difference, honestly, speaking for myself. I have a board, for example, in face to face therapy I write on it so that we maintain eye contact. I also have this technique: at the end of the session, just as I am closing the door and seeing the client off in the clinic, I say the final sentence and tell them to stay alone with it, which is actually an intervention.”*

*(CP3)*

*“I don’t think there is a major difference because there are certain core materials we use most often, the ones that have basically become part of our routine. Since we already have those materials as PDFs, we simply share those PDFs during online therapy in such situations.”*

*(SLT2)*

Differences reported only by the SLTs included that preparing individualized materials is more common in online settings (4), virtual games are used more frequently in online sessions (2), and book use is more often preferred in face-to-face therapy (1).

*“We need to translate everything into the online environment. Somehow it needs to be dynamic and engaging for children. Likewise, for an adult, if they are receiving therapy for aphasia or apraxia or dysarthria, you consider everything, such as ensuring the material is in a size and color they can see. However, in face to face settings, these materials are already printed, with the color, glossy paper, size, and image quality all considered. Even if we scan them for online use, sometimes we cannot fully achieve the same result.”*

*(SLT3)*

### 3.3. Advantages and Disadvantages of Online Therapy

**Figure 4:**
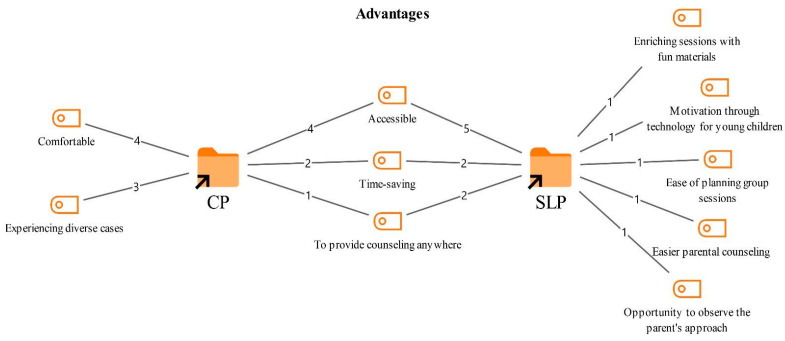
Codes related to the advantages theme.

**Figure 5:**
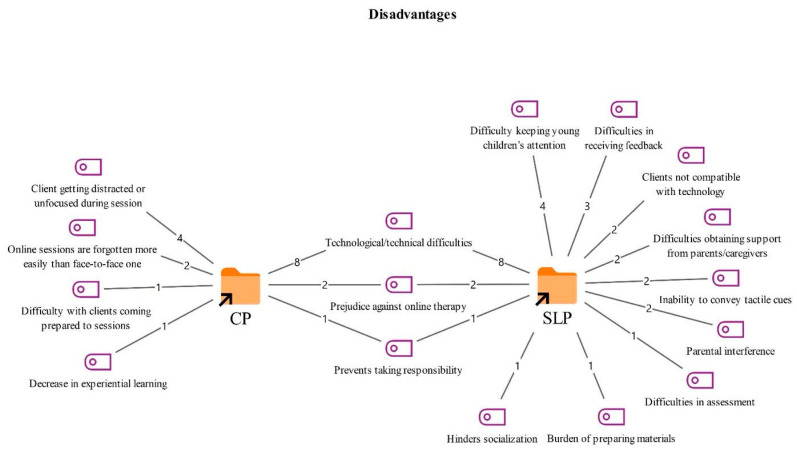
Codes related to the disadvantages theme.

Participants were asked to describe the advantages and disadvantages of delivering online therapy. Based on their responses, the resulting codes and their frequencies are presented in Figure 4 and Figure 5.

As shown in Figure 4, the advantages reported by both professional groups included accessibility (9), time saving (4), and the opportunity to provide counseling services from anywhere (3). Participants stated that online therapy removes geographical barriers, offers flexibility, and enables savings in time.

*“Especially people living abroad, in Europe, who may not receive sufficient benefit due to cultural codes apply, or people reach us from different parts of the country, from Anatolia, because popular and well known therapists live in big cities. This is, of course, an advantage.”*

*(CP5)*

*“For me, it has also made things easier because yes, sometimes I need to travel outside the city, it could be for a conference or a vacation. If we can plan it mutually, then I do not need to cancel sessions; I can still prepare and continue online.”*

*(SLT2)*

Advantages mentioned only by the CPs included that online therapy can be more comfortable (4) and that it provides opportunities to work with a wider range of cases (3). CPs emphasized that being able to meet with clients from different cities and even different countries is advantageous in terms of case diversity.

*“I had the chance to see a lot of clients. At university, I generally worked with the 18 to 23 age group, but in online therapy my age range expanded. I had the chance to connect with clients from different professions and living different lives.”*

*(CP2)*

The SLTs also reported that online therapy offers additional opportunities in certain respects. These advantages included the possibility of enriching sessions with engaging materials (1), technology serving as a motivational factor for young children (1), and easier planning of group sessions (1). In addition, participants noted that online therapy facilitates parent counseling (1) and provides an opportunity to observe how parents interact with the child in the home environment during sessions.

*“Besides that, as an advantage, I mentioned that using materials in the environment can also be a source of motivation, so we can add that as well. For example, I use drawing games a lot, such as having children draw and then guessing… But I think decorating an online session with these tools allows us to make greater use of the benefits of technology. The child also becomes very motivated.”*

*(SLT4)*

As shown in Figure 5, the disadvantages reported by both professional groups primarily included technological and technical problems during sessions (8) and reduced responsibility taking on the part of clients (2). Participants noted that technical issues such as internet connectivity and audio or video quality can disrupt the therapy process. They also suggested that online therapy may reduce clients’ active engagement in the therapeutic process.

*“Technical problems can occur, there can be connection problems. Disconnection, freezing, lighting issues, not being able to see the client clearly, for example the angle of the light can make it difficult to see the client’s facial expressions.”*

*(CP5)*

*“For adolescents, for example, getting up and coming here, coming to a place, coming to the clinic is actually a responsibility. But sometimes my adolescent clients, not to say they are lazy, but they can avoid these responsibilities even when it comes to simply logging into online sessions. Very casually it can be like, ‘Teacher, I forgot the session,’ and things like that can happen.”*

*(SLT2)*

Disadvantages mentioned only by the CPs included online sessions being forgotten more often than face-to-face sessions (2), reduced experiential learning (1), difficulties in clients coming to sessions psychologically and mentally prepared (1), and challenges in establishing trust (1). CPs reported that clients may remember online therapy less consistently and that their commitment to sessions can decrease.

*“I don’t know if it’s related to the easy accessibility of online therapy, but patients forget. Of course, forgetting a session can happen face to face too, but online it can be perceived as less serious and then there is more forgetting, although it varies from person to person. There is a quicker tendency to postpone. You need to get into that route. When the person is not ready for therapy, they can quickly slip into postponing and forgetting… If the person is coming to therapy, our preference is for them to go through that travel process, to come to us with thoughts forming on the way.”*

*(CP3)*

*“Ensuring the client’s safety and gaining their trust is a bit more difficult online because you can only see the camera angle; you don’t know what is behind it, who is there. This creates a disadvantage for the client. Doing it in a clinic room or an office might be similar in that sense.”*

*(CP1)*

The SLTs, in particular, drew attention to technical and cognitive factors such as difficulty obtaining feedback from older clients in front of a screen (3), challenges in working with younger age groups due to limited attention spans (2), and difficulties conducting sessions with clients who are not comfortable with technology (2). They also highlighted additional disadvantages, including the increased workload associated with preparing online specific materials (1), online therapy limiting clients’ opportunities for socialization (1), and parental interference during sessions (1).

*“For example, I show something, I’m doing semantic work. I say, ‘Which one is the book?’ I want them to point to the book. They point to something else, but in that moment I can’t see whether it’s correct or not. The person next to them needs to check and tell me, but sometimes they don’t give me feedback about whether it’s correct or not.”*

*(SLT3)*

*“If the child is very young, for example under the age of 4 or 5, as young as pre preschool, then online therapy is of course not very efficient because the child cannot sit in front of the computer anyway. In such cases, we rely on support from families… Especially for older groups such as people with aphasia or similar conditions, getting up and coming to a session, coming to a place, can also be a social activity for them… But in online therapy, they are still just sitting in their own rooms.” *

*(SLT2)*

### 3.4. Coping Strategies for Challenges in Online Therapy

**Figure 6:**
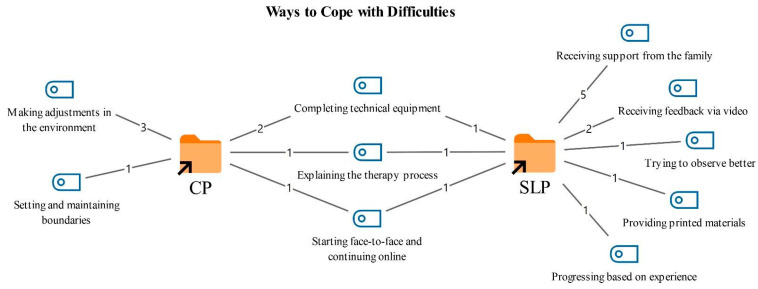
Codes related to coping strategies for challenges.

Participants were asked about the ways they cope with challenges during online therapy; the resulting codes and their frequencies are presented in Figure 6.

As shown in Figure 6, coping strategies used by both professional groups included ensuring that technical equipment is in place (3), explaining the therapy process to clients at the beginning of sessions (2), and starting with face-to-face sessions before gradually transitioning to online therapy (2). Participants reported that having adequate technical resources and clearly explaining the process to clients facilitates adaptation to online therapy. In addition, some participants stated that it can be more effective to help clients become familiar with therapy in person before moving to an online format.

*“If the electricity or internet goes out, I switch to mobile data… And when the electricity goes out, everyone loads onto mobile data, so the signal strength is affected naturally… Most of the time I use mobile internet. If I know in advance, for example it happened to me, there was an internet outage that lasted three months, three months… How did I cope with that? I stayed at my friend’s house.”*

*(CP2)*

*“I try to explain it as much as possible in a way that the caregiver can understand… That’s why I also shape my own language a bit according to the level of understanding of both the patient and the caregiver.”*

*(SLT5)*

*“If possible, doing a few face to face sessions before starting online therapy improves the quality of online therapy. Seeing and experiencing the client face to face a little, observing their facial expressions, gestures, and emotional state in a close relationship, allows us, even when we are in front of a screen, to feel how they respond and where, even if we cannot see every reaction clearly.”*

*(CP5)*

Coping strategies reported only by the CPs were primarily related to arranging the physical environment in which sessions took place (3). Participants described providing guidance so that clients could attend sessions under similar physical conditions, and they reported setting boundaries to reduce distractions during sessions and encouraging adherence to these rules (1).

*“For my part, I try to conduct sessions in the office. In my clinic, in fact not at the desk but in my armchair, I try to do it as if the client is in front of me. Of course, sometimes I also do it at home or elsewhere. Compared to those, I think doing it in the office is more effective.”*

(*CP1)*

*“You need to set boundaries from the beginning. In every part where they do not comply, you remind them of these boundaries again and again. Of course, the therapist also needs to comply with these boundaries… The therapist has a duty to protect the client and should take responsibility for what is in the client’s best interest.”*

*(CP3)*

In contrast, the SLTs frequently reported using strategies such as obtaining support from family members during sessions and receiving feedback via video (2). Additional approaches included asking clients to obtain printed materials and using them during sessions (1), and flexibly adapting the course of therapy according to the client’s needs (1).

*“If there is a problem, I get support from someone who knows, or from the family. For example, if I need to provide a reinforcer to the child during a speech sound disorder or fluency session, I ask the family.”*

*(SLT2)*

*“Sometimes I normally do not ask families to buy books or printed materials. I provide photocopies or printouts, I am talking about the clinic setting. Online, since we cannot do that, instead I can ask them to purchase those materials so that they have them and can work better. That might be an addition compared to face to face.”*

*(SLT4)*

*“It is also about me gaining experience. Because I have been through these things, I can think about the next session. Because I have experienced it before, I have a Plan B. But in the beginning, since we had not experienced it, you end up having to find a solution in the next session or the one after. That is how I cope, as we gain experience, we cope.”*

*(SLT3)*

### 3.5. Professional Implications of Online Therapy

**Figure 7:**
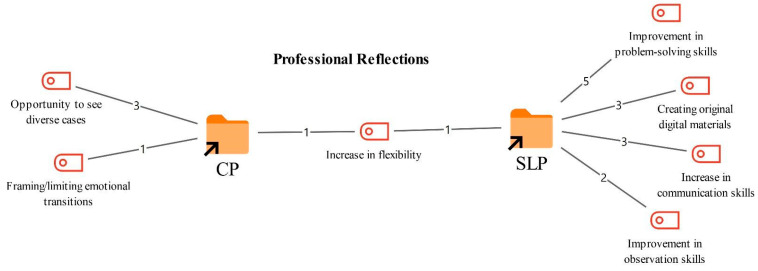
Codes related to the professional implications theme.

Participants were asked about how delivering online therapy has affected their professional practice as therapists. Based on their responses, the resulting codes and their frequencies are presented in Figure 7.

As shown in Figure 7, the most prominent gain reported by both professional groups was increased flexibility in managing the process and in decision making (1). Participants noted that online therapy requires greater adaptation in technical and logistical terms, and that this has helped strengthen their skills in managing therapeutic processes.

*“Because I work relationally as a therapist, the relationship I establish with the person in front of me is important for every therapist, of course, but as a couples therapist I needed to be able to provide flexibility in their life. This is part of my therapist side, and I gained that flexibility… Online work is part of this. No matter how much distance there is, no matter how much cultural difference there is, you need to be able to manage that collaboration and the therapy process from a flexible position.”*

*(CP3)*

Professional gains mentioned only by the CPs included strengthening professionally by having opportunities to work with a wider range of cases (3). CPs stated that they could reach a broader client population online, including different age groups and a variety of psychological concerns, and that this positively contributed to their professional development. They also reported developing skills in framing and containing emotional exchanges, noting that emotions can sometimes appear more salient on screen during sessions.

*“Different case experiences, different cultures for instance, I have been working in Istanbul for 20 years, but the clients I saw were always people who were here, so we work with cultures we know. … We see that the concepts of normal and abnormal can vary by culture. Pathologies also change in this sense… Working with someone from China or Europe was eye opening and instructive, especially in terms of experiencing different cultural codes.”*

*(CP5)*

*“I think I have improved in terms of conveying emotional transitions, I guess. Reading facial expressions, reading body language, and so on… In online therapy, it is harder to frame things and set boundaries. At this point I keep it tighter. That can be developmental, perhaps.”*

*(CP1)*

In contrast, the SLTs emphasized that the online therapy process provided opportunities to develop their problem solving skills (5). They also reported gaining competence in preparing original, client tailored digital materials (3), strengthening their communication skills (3), and improving their observational skills (2).

*“I think my problem solving skills have improved the most. Because of the internet dropouts I mentioned, or the client losing focus in that moment especially if it is a child, but even if it is an adult someone may enter the room and distract them. A problem emerges there. As the therapist, I need to take responsibility to solve it… In terms of observation skills, asking families for videos frequently, or if it is an adult asking them to record themselves while doing their exercises and send it, watching those videos second by second, rewinding, and checking again and again, I think it develops me like a detective. Because what we might lose during the live session becomes recorded this time, and it makes me a better observer.” *

*(SLT4)*

*“Definitely materials, in terms of materials, searching a lot, cutting and editing, using PowerPoint and animations has helped me a great deal. I search for foreign resources a lot on the internet, looking at what they have done and what I can do. It helps me a lot regarding materials, and I think I have developed myself professionally as well.”*

*(SLT3)*

### 3.6. Improvements Needed to Enhance Client Gains in Online Therapy

**Figure 8:**
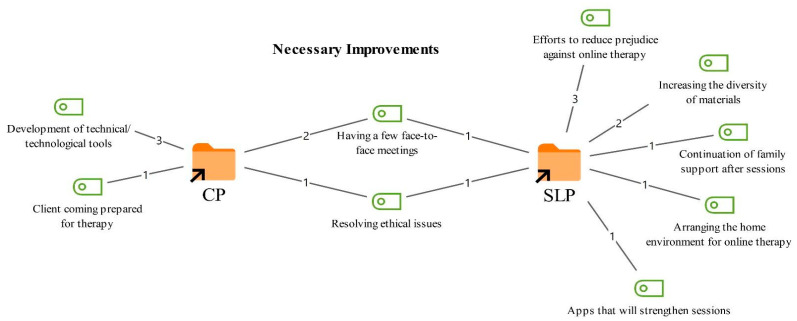
Codes related to the improvements needed theme.

Participants were asked which factors should be improved to increase clients’ gains in online therapy. Their responses and the corresponding frequencies are presented in Figure 8.

As shown in Figure 8, improvement suggestions reported by both professional groups included incorporating a few face-to-face meetings between online sessions (3) and addressing ethical issues (2). Participants noted that a fully online process may be insufficient in some cases and that adding periodic face-to-face meetings could enhance clients’ gains throughout therapy. They also emphasized that online therapy should be grounded on a more robust ethical framework.

*“We invite clients who receive online therapy to face to face sessions. If possible, doing a few face to face sessions before starting online therapy increases the quality of online therapy. Seeing and experiencing the client face to face a little, observing their facial expressions, gestures, and emotional state in a close relationship, allows us, even when we are in front of a screen, to feel how they respond and where, even if we cannot see every reaction clearly.” *

*(CP5)*

*“It could be improved in terms of security through certain systems as well. Some websites call for advertising, saying you can deliver online therapy through them, but when I look at how much attention they pay to ethical issues, they say it is only online and it is up to you. They say they are affiliated with the Ministry of Health, but then they say ethical issues are up to you. It still does not feel reliable to me in that sense. I would say we need to improve in this area, in terms of trustworthiness.” *

*(SLT2)*

Recommendations mentioned only by the CPs included improving technical and technological tools (3) and developing practices to ensure that clients attend sessions psychologically and mentally prepared for therapy (1). CPs emphasized that strengthening the technical infrastructure in online therapy could increase session efficiency, and that specific applications or methods could be developed to help clients prepare more effectively for the therapeutic process.

*“Technical issues. In our country, the quality of internet connection is low. Technical devices could be used, even things like virtual reality methods today. By improving these, the realism of a one to one session environment could be integrated into online therapy. Only technical issues come to my mind.” *

*(CP5)*

*“I think the biggest problem is being ready for therapy. The more ready someone is, the more benefit they get. Abroad, for example, if someone does not attend their appointment, there is a sanction. When there is such a sanction online, it is as if it pushes the person to start therapy. Of course, this is also related to face to face therapy, but it applies to both.”*

*(CP3)*

Recommendations reported only by the SLTs included conducting initiatives to reduce bias toward online therapy (3), increasing the diversity of materials (2), maintaining family support after sessions (1), arranging the home environment for online therapy (1), and developing digital tools or mobile applications to strengthen sessions (1). SLTs emphasized that diversifying materials, particularly when working with children, may support the therapy process, and that families’ more active involvement is important.

*“Focus could be placed on digital materials. I think awareness raising efforts could also be carried out for caregivers and for our other colleagues who do not provide online therapy. Because we therapists also start out somewhat biased at first… Because the more comfortable and at ease we feel, the better we manage the process.”*

(*SLT5)*

*“Also, in the online environment, whether for children or adults, caregivers need to engage with the process much more effectively. If homework is assigned, they need to support homework; if a video is requested, they need to support the video; or they need to be able to sit alongside the client for those 40 min. Because in face to face settings, the person does not need to wait there for 40 min; they can create their own space outside with their phone, as long as the parents do not come in. But in the online environment, I especially expect them to take an active role almost as much as the client, both for technological reasons and to manage the child in the moment or, if it is an adult, to provide feedback to me in any situation.”*

*(SLT3)*

### 3.7. Ethical Issues in Online Therapy and Their Solutions

While both groups emphasized digital privacy, a distinct divergence emerged regarding physical ethics. CPs focused predominantly on ‘relational boundaries’ and ‘therapeutic frame maintenance’ in a virtual space. Conversely, SLTs highlighted ‘physical safety’ as a paramount ethical concern, particularly regarding the risk of aspiration or choking during tele-rehabilitation for swallowing disorders, where the therapist cannot intervene physically.

**Figure 9:**
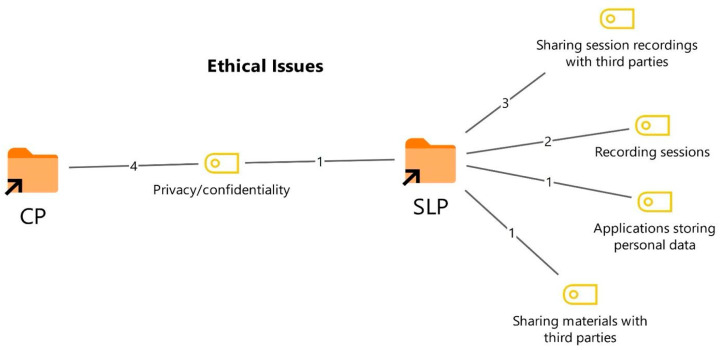
Codes related to the ethical issues theme.

**Figure 10:**
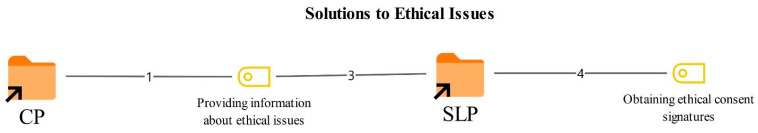
Codes related to solutions for ethical issues.

Participants were asked about the most important ethical issues they encounter during the online therapy process and how these issues could be addressed. The resulting codes and their frequencies are presented in Figure 9 and Figure 10.

As shown in Figure 9, the CPs identified confidentiality and security as the most significant ethical issue in online therapy (4). CPs emphasized that protecting the confidentiality of online sessions, ensuring the secure storage of clients’ personal information, and preventing data breaches are among the most critical ethical concerns.

As shown in Figure 10, the solution reported by both professional groups was informing clients before therapy about ethical issues (3). Participants emphasized the importance of clearly informing clients or families about confidentiality, recording practices, and data security in online therapy.

*“Perhaps it is about trustworthiness, whether we can ensure it or not. In face to face therapy, we maintain confidentiality, after all. But if someone else is present in the environment, I am intervening with one person, yet everyone may interpret it differently. This undermines the client’s adaptation.”*

*(CP3)*

The SLTs focused more on session recordings and the sharing of materials with third parties. The most frequently reported ethical issues included unauthorized sharing of session recordings with third parties (3), sessions being recorded without the client’s knowledge or consent (2), and concerns about video conferencing applications storing personal information (1). In addition, participants mentioned ethical concerns such as families deciding whether to continue after a few sessions and discontinuing therapy prematurely (1), as well as the unauthorized sharing of therapy materials with third parties (1).

*“Ethically, I think this is also very important: we do not know whether the person on the other side, especially if they are an adult, is recording. Sometimes I explicitly say, ‘If you want to watch it later, you can record it,’ and then they feel a bit more comfortable and they want to record. Or I say, ‘Don’t record it yourself, I can record it and send it to you,’ because I don’t know who they might show it to.”*

*(SLT2)*

*“Another issue is actually about keeping recordings. We sometimes use Zoom, Teams, or even WhatsApp video calls, for example in certain exercises or stuttering street activities. I have used three or four applications so far. But we do not really have much information about how these platforms store personal data or how they dispose of the therapy records… Families insist that they want to receive sessions from me, they want to try, and if it doesn’t work they want to stop. They say, ‘Teacher, let’s try a few sessions, and if there is no progress we will go to someone else face to face.’ To resolve this ethical dilemma, I can use the pre session consultation.”*

*(SLT4)*

*“The ethical information form is, of course, somewhat different. At one point I add something there. In face to face therapy, the client comes to the clinic… Some even come with their coffee… But preparing coffee beforehand and joining the session that way is much more common. So I remember adding an item to the ethical information form stating that eating or drinking anything other than water is not appropriate, and that only drinking water is allowed. I send the forms online as well, which is another difference.”*

*(CP2)*

Solutions mentioned only by the SLTs highlighted obtaining written ethical consent/signatures (4). SLTs stated that written consent from clients or parents is necessary to prevent ethical issues, particularly regarding the sharing of session recordings, the use of therapy materials, and the involvement of third parties. In addition, they noted that explaining ethical issues in detail during pre-session consultations (1) could contribute to a healthier process.

*“To resolve this ethical dilemma, I can use the pre session consultation. I keep the initial consultation a bit longer and try to use my persuasion skills, I could say. Because I do not want to cause financial loss or waste of time; if I do not think it will be productive, I do not want to start with that person.”*

*(SLT4)*

*“Actually, I don’t know whether this has been done or not, but at the very beginning of my sessions I tell clients that they should not make video or audio recordings related to the therapy content. Then, if necessary, they should message me, but they should not record anything without permission.”*

*(SLT5)*

### 3.8. Recommendations for Colleagues Providing Online Therapy

**Figure 11:**
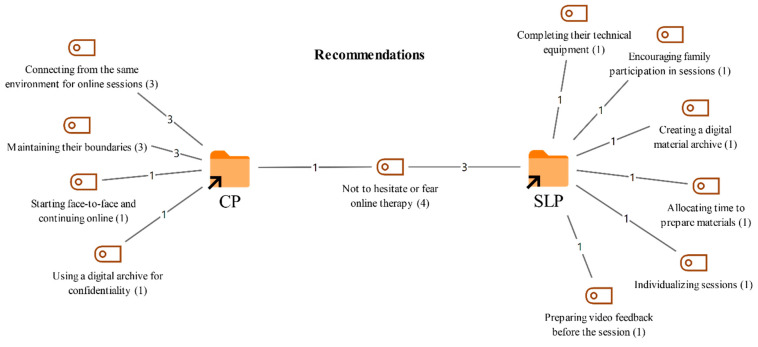
Codes related to the recommendations theme.

Participants were asked to share any recommendations they have for colleagues who provide online therapy. Their responses and the corresponding frequencies are presented in Figure 11.

As shown in Figure 11, the most prominent recommendation emphasized by both professional groups was that colleagues should not hesitate or be afraid of providing online therapy (4). Participants stated that online therapy can be as effective as face-to-face therapy and that colleagues should be more willing and confident in adapting to this format.

*“I often come across this in colleagues who are just starting out, in my interns, or even among my own friends. I think people are somewhat hesitant about online therapy, they are a bit afraid, wondering whether it will work and whether it will be as effective as face to face. I think they can be a bit more courageous about this. It can be tried, and after trying it can even become enjoyable. Sometimes it even provides convenience, especially in terms of accessibility, planning your own life, and not being tied to being physically inside the clinic.”*

*(SLT2)*

Recommendations mentioned only by the CPs included joining online sessions from consistent settings (3), which may provide clients with a more stable therapy experience, and maintaining clear professional boundaries (3), which was described as essential for sustaining a healthy therapeutic relationship. In addition, participants suggested starting therapy face to face before continuing online (1) to facilitate adaptation, and using digital archiving to protect confidentiality (1).

*“In addition, it’s important to join sessions with a stable, consistent background. When I imagine myself sitting in the client’s chair, if my therapist’s background kept changing, it would inevitably catch my attention at the beginning of the session. I might be coming into the session with something completely different on my mind. If I went to a face to face session, I would see them in the same room every time and this wouldn’t be an issue. I can recommend these.”*

*(CP2)*

*“They should protect their boundaries well. I would recommend that they draw a strong frame, because they will continue with that frame afterwards as well. Since conditions and situations can vary during the session, I would recommend that they make appropriate interventions regarding ethical and boundary issues and define their lines very clearly. I would advise them not to change their own physical conditions.”*

*(CP1)*

*“If possible, before starting online therapy, individuals should attend face to face therapy if there is an opportunity. If not, inviting them for a face to face session after six months to one year increases the quality of therapy.”*

*(CP5)*

*“If you’re working from home, and you live with other people, how will you store client files? You archive client notes and so on, but how sure can you be about confidentiality if you don’t have a locked cabinet? Maybe you can obtain one. So as a recommendation, if they can get used to it, taking notes on a tablet and transitioning to that could be easier. For someone working online from a home office, it provides convenience for archiving.”*

*(CP2)*

In contrast, the SLTs emphasized that, for online therapy to be efficient, technical equipment should be adequately set up (1) and that supporting family involvement in sessions (1) is especially important in therapies conducted with children. They also recommended creating a digital materials archive (1), allocating time to prepare materials specifically for online sessions (1), and individualizing sessions according to clients’ needs (1). In addition, preparing video based feedback before sessions (1) was suggested as a useful strategy for monitoring client progress.

*“They should have good lighting and a good environment, and definitely not overlook technical details. If computer cameras are not sufficient, getting a new camera, getting a new microphone, these are important, I think… If it’s a child, I recommend making it a condition that the family remains in the session for the full 40 min… Also, especially regarding materials, I would recommend keeping a digital archive. I suggest creating folders for each disorder, and having digital versions of whatever they might need in face to face sessions in those folders, because sometimes there is a moment when you suddenly need an exercise, like ‘oh, let’s try this too’… In that moment, I recommend having materials that are easily accessible from that library.”*

*(SLT4)*

*“They need to allocate time. For example, before the session, we are very busy on Saturdays, we quickly pick materials and jump in, but for online, especially, I want some methods like Palin. Watching at least three videos totaling 15 to 20 min, commenting on them, and then developing materials on top of that takes a long time.”*

*(SLT3)*

*“I think one can be more prepared. Because the more I prepare for therapy, the safer I feel… When you can share your screen and say, ‘I prepared something like this for you,’ especially when you do it in a personalized, client specific way, one of the tactics I use most is to ask the family for a photo of a toy the child loves or a moment they experienced and add it to the presentation. Children are surprised by this. When we approach them in a more individual, client specific way, it even feels like magic to them.”*

*(SLT2)*

## 4. Discussion

The findings of this study provide a comprehensive overview of how CPs and SLTs navigate the transition to teletherapy. A synthesis of the major themes, professional similarities, and key divergences is presented in Table 2. As illustrated in the summary table, while both professions successfully maintain the therapeutic alliance and share common technical hurdles, they diverge significantly in their focus on ethical safety and material-mediated engagement. The following subsections interpret these findings in relation to the existing literature and theoretical frameworks.

### 4.1. Comparative Insights into the Therapeutic Alliance

This study aimed to develop an in depth understanding of SLTs’ and CPs’ experiences with teletherapy and to compare these experiences across the two professional groups. Although both groups viewed teletherapy as an effective and feasible service delivery model, they also highlighted ethical issues, challenges, and disadvantages. Most participants reported that teletherapy is similar to face-to-face therapy in many respects. This finding is consistent with prior work indicating that therapist client alliance in online therapy can be comparable to that in face-to-face contexts [38]. The findings further suggest that online therapy was not framed as a secondary or lower quality intervention; rather, it was described as a format that can enhance professional flexibility and access to services under specific conditions. This interpretation aligns with previous literature [9,16,17,45]. In particular, CPs noted that online therapy can improve accessibility for individuals with demanding schedules [45].

At the same time, some therapists reported that the therapeutic bond and emotional mirroring were established more strongly and more rapidly in face-to-face sessions than in online sessions. This finding is also consistent with studies reporting stronger or more immediate relational processes in face-to-face modalities [46]. In addition, CPs drew attention to the possibility that the quality of the therapeutic relationship may vary depending on case characteristics, whereas SLTs emphasized the role of parent mediated processes in online delivery. This difference may reflect the fact that SLTs in this study more frequently worked with pediatric populations and routinely involved families in the intervention process. The literature frequently notes that factors such as reduced trust, decreased communication adequacy, and disruptions in the naturalness of interaction may negatively affect the therapeutic relationship [11,47,48,49]. Building on this, it is important to acknowledge that families’ mediation during teletherapy sessions, particularly in child focused and family based interventions, should be considered among the factors shaping the therapist client relationship, alongside the influence of technological devices on relational dynamics [38].

### 4.2. Balancing Advantages and Practical Challenges of Teletherapy

When examining the advantages and disadvantages of online therapy, both professional groups emphasized accessibility, time saving, and the possibility of delivering services from anywhere. For the SLT group, teletherapy specific advantages included the opportunity to observe the home environment, the facilitation of parent counseling, and the feasibility of planning group sessions. These findings align with prior research indicating that online psychological counseling is often viewed as advantageous due to improved accessibility and cost effectiveness, the ability to reach clients across different cities and countries, the option to continue sessions while traveling, and the feasibility of organizing therapy within demanding schedules [50,51].

Technical problems and reduced client responsibility taking were identified as shared disadvantages across both groups. In contrast, adaptation difficulties when working with specific populations, such as young children and geriatric individuals, were more salient for the SLT group. Technical and technological problems have also been highlighted across multiple studies [19]. While CPs pointed out that online sessions may be more easily forgotten, SLTs emphasized disadvantages such as difficulty obtaining real time feedback from clients during sessions, and challenges related to technological adaptation when working with young age groups and geriatric individuals. In addition, the literature has noted further disadvantages, including that therapy conducted in the home environment may make it harder for both clients and therapists to enter a therapeutic mindset, may increase environmental distractions, and may be hindered when the home setting is not arranged appropriately for therapy [20].

### 4.3. Ethical Landscapes and Case Appropriateness

The literature indicates that one of the most fragile and disadvantageous aspects of online therapy for CPs concerns ethical risks, particularly confidentiality and privacy. In online counseling, the inability to ensure privacy in the client’s setting, interruptions within the home, the possibility that third parties may listen to sessions, and uncertainties regarding data security are consistently described as core ethical issues that can threaten the therapeutic process [52]. Zeren identified technical disruptions and the lack of transparency in platforms’ data storage policies as major areas of ethical risk in online counseling [53]. These points align directly with the present study’s finding that CPs most frequently identified “confidentiality and trustworthiness” as the primary ethical concern in online therapy. Concerns such as being able to see only the camera angle, not knowing whether someone else is present in the room, and uncertainty about the security of digitally stored data can be viewed as concrete reflections of broader ethical debates in the literature as they manifest in clinical practice.

The ethical dimension is not limited to confidentiality and technical infrastructure; the question of which cases are appropriate for online therapy is also strongly framed as an ethical issue in the literature. Andersson [54] emphasized that online therapy should be restricted in situations such as working with children under the age of 18, severe psychiatric disorders, psychotic presentations, and substance or alcohol dependence. Similarly, Tuzgöl [55] noted in a review that online interventions may have limited effects in cases involving developmental disorders and in presentations where reality testing is compromised. From the perspective of speech and language therapy, teletherapy has been considered particularly suitable in areas such as voice and fluency disorders [18]. Another study concluded that teletherapy can be effective across a wide range of diagnoses, including neurogenic disorders and swallowing disorders [31]. In the literature, CPs have also highlighted challenges such as conducting teletherapy in distracting environments and the potential unsuitability of teletherapy for severe psychiatric cases [45].

### 4.4. Clinical Adaptations, Professional Growth, and Future Directions

Despite these disadvantages, both professional groups reported developing various strategies to cope with challenges encountered in online therapy. Participants described solutions such as ensuring that their technical equipment was adequate, explaining the therapy process to clients in detail, and beginning therapy face to face before continuing the process online. While CPs tended to manage challenges primarily through arranging the physical setting in which sessions took place, SLTs commonly relied on family support and obtained feedback via video. The literature similarly emphasizes that caregivers should place greater importance on supporting clients’ participation during teletherapy sessions [56].

All CPs reported using similar techniques and materials in teletherapy as in face-to-face sessions. Among SLTs, some also indicated that they used comparable techniques and materials; however, several therapists drew attention to the difficulty of not being able to use tactile cues in online settings. They further emphasized the importance of family support and noted that preparing individualized materials for online therapy can be more time consuming compared to face-to-face therapy. Both professional groups reported that teletherapy increased their flexibility. CPs emphasized that online delivery offered opportunities to work with a wider range of case types. In contrast, SLTs highlighted several professional benefits, including improved problem solving skills, greater competence in preparing materials, enhanced communication skills, and strengthened observational skills. Compared with earlier research, a distinctive contribution of the present study is its explicit focus on perceived development in therapists’ clinical skills as an outcome of teletherapy practice.

With regard to ethical issues, CPs identified confidentiality and security as the most important ethical problem, whereas SLTs focused more on issues such as third party sharing of session recordings, recording without consent, and the security of therapy materials. As solutions, both groups emphasized the importance of informing clients about ethical issues before therapy, while SLTs additionally underlined the need to obtain written ethical consent. Consistent with these findings, the literature highlights concerns about the risk of session content being shared, the protection of confidentiality, and the central role of informed consent processes in teletherapy [19,21]. Both professional groups recommended integrating face-to-face sessions into the online process at certain intervals and implementing improvements to address ethical problems. The SLTs highlighted the need to increase awareness raising initiatives to reduce biases about therapeutic gains and to expand the diversity of materials. In contrast, the CPs emphasized that enhancing teletherapy effectiveness requires a stronger focus on technological advancements. These recommendations for improving client gains also provide a practical roadmap for strengthening teletherapy sustainability and therapeutic effectiveness.

Finally, both the literature and the present findings underline that the advantages and disadvantages of online therapy coexist, and that it is not an ethically “black and white” domain. Barnett [57] and Poyrazlı and Can [58] noted that online therapy offers an indispensable opportunity for clients facing constraints of time and place, while technical problems, limited oversight, and the possibility of unauthorized providers delivering services may weaken the ethical framework. The present findings further illuminate these issues from the perspectives of two professional groups. Specifically, the ethical concerns emphasized by CPs, which centered more on the therapeutic frame, boundaries, and privacy, and those emphasized by SLTs, which focused more on materials use, family involvement, and the circulation of recordings, help make visible the profession specific features of online service delivery.

## 5. Limitations

The study has several limitations that should be considered when interpreting the findings. First, the small sample size (N = 10) and the geographic concentration of participants, primarily in Istanbul, limit the broader transferability of the findings to rural or more diverse regions across Türkiye. While a sample of ten is consistent with the standards of descriptive phenomenology, it nonetheless restricts the scope of the conclusions. Second, the use of convenience sampling may have introduced a self-selection bias, as practitioners who are already more comfortable with or positively inclined toward teletherapy were likely more motivated to participate in the study.

Third, a significant imbalance in teletherapy exposure existed within the sample, partly due to the differing developmental stages of the two professions in Türkiye. For instance, while one participant (CP5) reported extensive experience with over 2000 online cases, others had substantially less exposure, which may have influenced the varying depth and nuance of certain reflections.

Fourth, although “profession-matched” interviewing was intentionally employed to build rapport and facilitate a shared professional language, it may have introduced “interviewer effects” or “insider bias.” This positionality could potentially result in certain professional assumptions being left unprobed due to the shared background between the researcher and the participant.

Finally, the two professional groups differed in their predominant caseloads-CPs primarily worked with adults, whereas SLTs focused on pediatric populations. Accordingly, some observed differences may reflect variations in client demographics rather than profession-specific factors alone. Furthermore, the predominantly female sample, while reflective of the demographics of these fields, may overlook the unique perspectives of male practitioners. Future research would benefit from utilizing longitudinal designs and comparing larger, more gender-balanced groups with closely matched case profiles and more diverse geographic representation.

## 6. Conclusions

The findings of this study indicate that teletherapy has transitioned from a crisis-response substitute into a permanent and standardized modality within the Turkish healthcare landscape. This study reveals that the digital transposition of therapy is deeply profession-specific: whereas clinical psychologists focus on preserving the therapeutic frame, emotional synchrony, and relational boundaries, speech and language therapists navigate specific challenges related to physical safety-such as aspiration and choking risks-and the complexities of material-mediated pediatric engagement. These findings demonstrate that generic telehealth protocols are insufficient for diverse clinical needs.

Consequently, there is a critical need to formally integrate standardized, profession-specific teletherapy modules into undergraduate health sciences curricula to equip future practitioners with necessary digital competencies. Furthermore, professional associations and health authorities should develop clinical guidelines that address the unique technical and ethical requirements of each discipline. Future research should prioritize longitudinal designs to evaluate long-term clinical outcomes and include larger, geographically diverse samples to enhance the transferability of the findings. Ultimately, the successful evolution of digital health services hinges on recognizing these professional nuances to ensure both therapeutic efficacy and ethical safety in an increasingly virtual clinical environment.

## Figures and Tables

**Table 1:** Participant characteristics.

Participant	Age	Gender	Education Levels	City	Years of Professional Experience	Client Population	Number of Online Therapy Cases
SLT1	28	Female	Msc	İstanbul	5	Aphasia, speech sound disorder, voice disorder, fluency disorder, family counseling	300
SLT2	26	Female	Msc	Eskişehir	5	Language disorder, speech sound disorder, fluency disorders, motor speech disorders, voice disorder	200
SLT3	29	Female	Msc	Mersin	5	Preschool-aged children, adolescents, adults; fluency disorders, delayed language and speech, articulation disorder	100
SLT4	25	Female	Msc	İstanbul	4.5	Children, adults; developmental language disorder, delayed language and speech, stuttering, speech sound disorder, voice disorder	250
SLT5	27	Female	Msc	İstanbul	5	Aphasia, speech sound disorder, fluency disorder	120
CP1	36	Female	Msc	İstanbul	13	Adults; depression, anxiety disorders, relationship issues, couples therapy	100
CP2	26	Male	Msc	İstanbul	3	Adults; anxiety, coping with stress, adjustment to life events	300
CP3	32	Female	Msc	İstanbul	8	Adults, couples; relationship issues, communication problems, emotional conflicts	170
CP4	28	Female	Msc	İstanbul	3	Children, families, adults; parent counseling, emotion regulation difficulties, relationship issues	170
CP5	42	Male	Msc	İstanbul	20	Adults; mood problems, grief and bereavement processes, life transitions	2000

**Table 2:** Summary of the eight major themes and cross-professional comparison.

Major Themes	Key Findings	Professional Similarities	Professional Differences
1. Transition to Teletherapy	COVID-19 pandemic acted as a primary catalyst for adopting online practice.	Initial skepticism followed by rapid adaptation across both groups.	CPs: Focused on ensuring continuity of long-term therapy. SLTs: Focused on finding digital alternatives for physical materials.
2. Therapeutic Relationship and Alliance	Therapeutic alliance can be successfully established and maintained online.	Both groups emphasize active listening and verbal mirroring to build rapport.	CPs: Prioritize emotional “presence” and verbal depth. SLTs: Rely on screen-sharing and interactive activities to engage clients.
3. Assessment and Intervention Techniques	Traditional face-to-face techniques were modified for the digital environment.	Use of digitalized materials and shared screens for session flow.	CPs: Use of verbalizing non-verbal cues (e.g., “I see you looking away”). SLTs: High reliance on gamification and digital tools for pediatric cases.
4. Perceived Advantages	Increased accessibility, time efficiency, and flexibility for both therapists and clients.	Reduction in transportation barriers and increased attendance rates.	CPs: Noted clients feel more comfortable sharing in their own space. SLTs: Highlighted the shift toward family-centered “coaching” models.
5. Challenges and Disadvantages	Technical glitches and limited physical interaction are the main hurdles.	Internet connectivity and audio-visual lag disrupt the flow of sessions.	CPs: Difficulties in perceiving subtle somatic cues or silences. SLTs: Challenges in physical assessment and lack of tactile feedback.
6. Ethical Issues	Privacy and data security are the paramount ethical priorities in digital spaces.	Both groups strictly follow informed consent and secure platform protocols.	CPs: Focus on managing virtual “relational boundaries.” SLTs: Highlight physical safety risks (e.g., choking/aspiration) in swallowing therapy.
7. Coping Strategies	Practitioners develop creative workarounds to manage digital limitations.	Use of alternative communication (e.g., phone calls) during tech failures.	CPs: More explicit verbal expressions of empathy. SLTs: Intensive involvement of parents as “co-therapists” in the home.
8. Future Perspectives	Teletherapy is viewed as a permanent and standardized intervention modality.	Preference for hybrid models and a call for formal teletherapy training.	CPs: Emphasize reaching underserved rural populations. SLTs: Advocate for the development of profession-specific digital assessment tools.

## Data Availability

The raw data supporting the conclusions of this article will be made available by the authors on request.

## Supplementary Materials

The following supporting information can be downloaded at: https://www.mdpi.com/article/10.3390/healthcare14060716/s1, Supplementary Material S1: Interview Questions.

## References

[1] Brennan D., Tindall L., Theodoros D., Brown J., Campbell M., Christiana D., Smith D., Cason J., Lee A. (2010). A blueprint for telerehabilitation guidelines. Int. J. Telerehabilit..

[2] Chakrabarti S. (2015). Usefulness of telepsychiatry: A critical evaluation of videoconferencing-based approaches. World J. Psychiatry.

[3] Thompson R.B. (2016). Psychology at a Distance: Examining the Efficacy of Online Therapy. Bachelor’s Thesis.

[4] Giordano C., Ambrosiano I., Graffeo M.T., Di Caro A., Gullo S. (2022). The transition to online psychotherapy during the pandemic: A qualitative study on patients’ perspectives. Res. Psychother. Psychopathol. Process Outcome.

[5] Rettinger L., Klupper C., Werner F., Putz P. (2023). Changing attitudes towards teletherapy in Austrian therapists during the COVID-19 pandemic. J. Telemed. Telecare.

[6] (2013). Joint Task Force for the Development of Telepsychology Guidelines for Psychologists. Guidelines for the practice of telepsychology. Am. Psychol..

[7] Keck C.S., Doarn C.R. (2014). Telehealth technology applications in speech-language pathology. Telemed. E-Health.

[8] Reynolds A.L., Vick J.L., Haak N.J. (2009). Telehealth applications in speech-language pathology: A modified narrative review. J. Telemed. Telecare.

[9] Theodoros D.G. (2008). Telerehabilitation for service delivery in speech-language pathology. J. Telemed. Telecare.

[10] ASHA (2025). Telepractice: Overview. https://www.asha.org/Practice-Portal/Professional-Issues/Telepractice/.

[11] Stoll J., Müller J.A., Trachsel M. (2020). Ethical issues in online psychotherapy: A narrative review. Front. Psychiatry.

[12] Barak A., Hen L., Boniel-Nissim M., Shapira N. (2008). A comprehensive review and a meta-analysis of the effectiveness of internet-based psychotherapeutic interventions. J. Technol. Hum. Serv..

[13] Andersson G., Cuijpers P. (2009). Internet-based and other computerized psychological treatments for adult depression: A meta-analysis. Cogn. Behav. Ther..

[14] Karyotaki E., Riper H., Twisk J., Hoogendoorn A., Kleiboer A., Mira A., Mackinnon A., Meyer B., Botella C., Littlewood E. (2017). Efficacy of self-guided internet-based cognitive behavioral therapy in the treatment of depressive symptoms: A meta-analysis of individual participant data. JAMA Psychiatry.

[15] Pamplona M.d.C., Ysunza P.A. (2020). Speech pathology telepractice for children with cleft palate in the times of COVID-19 pandemic. Int. J. Pediatr. Otorhinolaryngol..

[16] Brady A. (2007). Moving toward the future: Providing speech-language pathology services via telehealth. Home Healthc. Now.

[17] Doraiswamy S., Abraham A., Mamtani R., Cheema S. (2020). Use of telehealth during the COVID-19 pandemic: Scoping review. J. Med. Internet Res..

[18] Kocabıyık N.A., Demirci H. (2021). Examination of Speech and Language Therapists’ Views on Teletherapy Practices and Their Readiness for Change. Dil. Konuşma ve Yutma Araştırmaları Derg..

[19] Mashima P.A., Doarn C.R. (2008). Overview of telehealth activities in speech-language pathology. Telemed. E-Health.

[20] Tar-Mahomed Z., Kater K.A. (2022). The perspectives of speech–language pathologists: Providing teletherapy to patients with speech, language and swallowing difficulties during a COVID-19 context. S. Afr. J. Commun. Disord..

[21] Bayati B., Ayatollahi H. (2021). Speech therapists’ perspectives about using tele-speech therapy: A qualitative study. Disabil. Rehabil. Assist. Technol..

[22] Ruwaard J., Lange A., Schrieken B., Dolan C.V., Emmelkamp P.M.G. (2012). The effectiveness of online cognitive behavioral treatment in routine clinical practice. PLoS ONE.

[23] Trindade I.A., Guiomar R., Carvalho S.A., Duarte J., Lapa T., Menezes P., Nogueira M.R., Patrão B., Pinto-Gouveia J., Castilho P. (2021). Efficacy of online-based acceptance and commitment therapy for chronic pain: A systematic review and meta-analysis. J. Pain.

[24] Al-Alawi M., McCall R.K., Sultan A., Al Balushi N., Al-Mahrouqi T., Al Ghailani A., Al Sabti H., Al-Maniri A., Panchatcharam S.M., Al Sinawi H. (2021). Efficacy of a six-week-long therapist-guided online therapy versus self-help internet-based therapy for COVID-19-induced anxiety and depression: Open-label, pragmatic, randomized controlled trial. JMIR Ment. Health.

[25] Breuer L., Barker C. (2015). Online support groups for depression: Benefits and barriers. SAGE Open.

[26] Burgoyne N., Cohn A.S. (2020). Lessons from the transition to relational teletherapy during COVID-19. Fam. Process.

[27] Bayri H., Kabasakal H.Z. (2025). An overview of online psychotherapy and psychological counseling studies in Türkiye. J. Soc. Hum. Adm. Sci..

[28] Association of Marital and Family Therapy Regulatory Boards (2016). AMFTRB Best Practices Guidelines for the Practice of Teletherapy. https://amftrb.org/wp-content/uploads/2021/06/TS-Teletherapy-Guidelines-09.12.16.pdf.

[29] Lincoln M., Hines M., Fairweather C., Ramsden R., Martinovich J. (2014). Multiple stakeholder perspectives on teletherapy delivery of speech pathology services in rural schools: A preliminary qualitative investigation. Int. J. Telerehabilit..

[30] Cangi M.E., Toğram B. (2020). Stuttering therapy through telepractice in Turkey: A mixed method study. J. Fluen. Disord..

[31] Shahouzaie N., Gholamiyan Arefi M. (2024). Telehealth in speech and language therapy during the COVID-19 pandemic: A systematic review. Disabil. Rehabil. Assist. Technol..

[32] Cacciante L., Cieślik B., Rutkowski S., Rutkowska A., Kacperak K., Kuligowski T., Kiper P. (2021). Feasibility, acceptability and limitations of speech and language telerehabilitation during COVID-19 lockdown: A qualitative research study on clinicians’ perspectives. Healthcare.

[33] Chaudhary T., Kanodia A., Verma H., Singh C.A., Mishra A.K., Sikka K. (2021). A pilot study comparing teletherapy with the conventional face-to-face therapy for speech-language disorders. Indian J. Otolaryngol. Head Neck Surg..

[34] ter Huurne E.D., Postel M.G., de Haan H.A., DeJong C.A.J., Van der Palen J. (2015). Web-based treatment program using intensive therapeutic contact for patients with substance use disorders: Randomized controlled trial. J. Med. Internet Res..

[35] de Zwaan M., Herpertz S., Zipfel S., Svaldi J., Friederich H.-C., Schmidt F., Mayr A., Lam T., Schade-Brittinger C., Hilbert A. (2017). Effect of internet-based guided self-help vs individual face-to-face treatment on full or subsyndromal binge eating disorder in overweight or obese patients. JAMA Psychiatry.

[36] Lin T., Heckman T.G., Anderson T. (2021). The efficacy of synchronous teletherapy versus in-person therapy: A meta-analysis of randomized clinical trials. Clin. Psychol. Sci. Pract..

[37] Mohr D.C., Burns M.N., Schueller S.M., Clarke G., Klinkman M. (2013). Behavioral intervention technologies: Evidence review and recommendations for future research in mental health. Gen. Hosp. Psychiatry.

[38] Simpson S.G., Reid C.L. (2014). Therapeutic alliance in videoconferencing psychotherapy. Aust. J. Rural Health.

[39] Cangi M.E., Yaşa İ.C., Işıldar A. (2021). Preferences of speech and language therapists for telepractice in the COVID-19 pandemic and factors affecting their acceptance of the delivery model. Eur. J. Rehabil. Health Stud..

[40] Wilczewski H., Paige S.R., Ong T., Soni H., Barrera J.F., Welch B.M., Bunnell B.E. (2022). Providers’ perspectives on telemental health usage after the COVID-19 pandemic: Retrospective analysis. JMIR Form. Res..

[41] Creswell J.W., Poth C.N. (2018). Qualitative Inquiry and Research Design: Choosing Among Five Approaches.

[42] Oluka A. (2025). Phenomenological research strategy: Descriptive and interpretive approaches. F1000Research.

[43] Braun V., Clarke V. (2021). Can I use TA? Should I use TA? Should I not use TA? Comparing reflexive thematic analysis and other pattern- based qualitative analytic approaches. Couns. Psychother. Res..

[44] Creswell J.W. (2015). Educational Research: Planning, Conducting, and Evaluating Quantitative Research.

[45] Kotera Y., Ozaki A., Miyatake H., Tsunetoshi C., Nishikawa Y., Tanimoto T. (2021). Mental health of medical workers in Japan during COVID-19: Relationships with loneliness, hope and self-compassion. Curr. Psychol..

[46] Rotger J.M., Cabré V. (2022). Therapeutic alliance in online and face-to-face psychological treatment: Comparative study. JMIR Ment. Health.

[47] Norwood C., Moghaddam N.G., Malins S., Sabin-Farrell R. (2018). Working alliance and outcome effectiveness in videoconferencing psychotherapy: A systematic review and noninferiority meta-analysis. Clin. Psychol. Psychother..

[48] Békés V., Aafjes-van Doorn K. (2020). Psychotherapists’ attitudes toward online therapy during the COVID-19 pandemic. J. Psychother. Integr..

[49] Wind T.R., Rijkeboer M., Andersson G., Riper H. (2020). The COVID-19 pandemic: The “black swan” for mental health care and a turning point for e-health. Internet Interv..

[50] Harris B., Birnbaum R. (2014). Ethical and legal implications on the use of technology in counselling. Br. J. Guid. Couns..

[51] Mallen M.J., Vogel D.L., Rochlen A.B., Day S.X. (2005). Online counseling: Reviewing the literature from a counseling psychology framework. Couns. Psychol..

[52] Smith J., Gillon E. (2021). Therapists’ experiences of providing online counselling: A qualitative study. Couns. Psychother. Res..

[53] Zeren Ş. (2015). Face-to-face and online counseling: Client problems and satisfaction. Educ. Sci..

[54] Andersson G. (2010). The promise and pitfalls of the internet for cognitive behavioural therapy. BMC Med..

[55] Tuzgöl K. (2020). Ethics in Online Psychotherapy and Counseling. Turk. J. Integr. Psychother..

[56] Gallant A., Watermeyer J., Sawasawa C. (2023). Experiences of South African speech–language therapists providing telepractice during the COVID-19 pandemic. Int. J. Lang. Commun. Disord..

[57] Barnett J.E. (2005). Online counseling: New entity, new challenges. Couns. Psychol..

[58] Poyrazlı Ş., Ahmet C. (2020). Online Counseling: Ethical Guidelines, the COVID-19 Process, Suggestions. J. Sch. Couns..

