# Supplementary material for: “Therapy Beyond the Screen”: A Qualitative Exploration of Tele-Therapy Experiences Among Clinical Psychologists and Speech and Language Therapists
Source: Healthcare (Basel). 2026 Mar 11;14(6):716. doi: 10.3390/healthcare14060716 (PMC13025854; doi:10.3390/healthcare14060716)
Supplement: Supplementary file 1 [file healthcare-14-00716-s001.zip › healthcare-4186784-supplementary.pdf]

**Supplementary Material**

**Interview Questions**

1. Could you briefly describe your general procedures for delivering online therapy services?
2. How would you compare the therapeutic relationship you establish with clients during online therapy to that in face-to-face therapy?
3. Do the techniques you use in online therapy differ from those used in face-to-face therapy? If so, in what ways do they differ?
4. Do the materials you use in online therapy differ from those used in face-to-face therapy? If so, in what ways?
5. Could you discuss the advantages of providing online therapy services?
6. Could you discuss the disadvantages of providing online therapy services?
7. What are the most common challenges you encounter during the online therapy process?
8. When comparing online therapy with face-to-face therapy, how would you evaluate the management of the therapeutic process?
9. How do you cope with the challenges you encounter during the online therapy process?
10. How has the process of providing online therapy influenced your professional development as a therapist? Which of your skills have you observed to improve during this process?
11. In your opinion, which aspects of online therapy should be improved to enhance clients' outcomes or gains?
12. What are the most significant ethical issues you encounter in the online therapy process, and how do you manage them?
13. What recommendations would you offer to colleagues who provide online therapy services? In particular, which strategies would you recommend for those who are new to this practice?
14. Is there anything else you would like to add?
